# First-Trimester Hemoglobin-to-RDW Ratio in Pregnancies Subsequently Complicated by Preeclampsia: Association, Discrimination, and Clinical Interpretability in a Retrospective Cohort

**DOI:** 10.3390/jcm15166234

**Published:** 2026-08-12

**Authors:** Murat Haksever, Deniz Taşdemir, Selim Kandemir, Bekir Kahveci, Refaettin Şahin, Alp Koray Kinter, Savaş Özdemir, Atakan Tanaçan, Ismet Hortu

**Affiliations:** 1 Department of Perinatology, Sanliurfa Training and Research Hospital, 63040 Sanliurfa, Turkey; 2 Department of Gynecologic Oncology, Sanliurfa Training and Research Hospital, 63040 Sanliurfa, Turkey; 3 Department of Obstetrics and Gynecology, Sanliurfa Training and Research Hospital, 63040 Sanliurfa, Turkey; 4 Department of Obstetrics and Gynecology, Istinye University Faculty of Medicine, Liv Hospital, 34517 Istanbul, Turkey; 5 Department of Obstetrics and Gynecology, Recep Tayyip Erdogan University Faculty of Medicine, 53200 Rize, Turkey; 6 Department of Perinatology, Ankara Bilkent City Hospital, University of Health Sciences, 06018 Ankara, Turkey; 7 Department of Obstetrics and Gynecology, Ege University Faculty of Medicine, 35100 Izmir, Turkey

**Keywords:** first trimester, preeclampsia, hemoglobin-to-RDW ratio, red cell distribution width, hematologic biomarkers, retrospective cohort, discrimination

## Abstract

**Background/Objectives:** Preeclampsia is a heterogeneous hypertensive disorder of pregnancy, and accessible hematologic indices may reflect maternal physiologic differences before the clinical syndrome becomes evident. This study evaluated whether the first-trimester hemoglobin-to-red cell distribution width (Hb/RDW) ratio was associated with later documented preeclampsia and assessed its discrimination, relation to disease severity, and association with maternal–neonatal outcomes in a tertiary-care cohort. **Methods:** This retrospective cohort study included 224 singleton pregnancies managed at a tertiary referral center between January 2024 and December 2025. Data extraction, anonymization, and analysis were performed after ethics approval (Approval No. 72; 2 March 2026). Participants were categorized as preeclampsia-negative (*n* = 131) or preeclampsia-positive (*n* = 93). Preeclampsia and severe features were defined according to American College of Obstetricians and Gynecologists criteria. The Hb/RDW ratio was calculated from routine first-trimester complete blood count parameters obtained before clinical diagnosis or final outcome classification. ROC analysis, multivariable logistic regression, multicollinearity assessment, events-per-variable evaluation, calibration testing, and exploratory incremental discrimination analyses were performed. **Results:** The first-trimester Hb/RDW ratio was higher in pregnancies later complicated by preeclampsia. Single-marker ROC analysis showed moderate discrimination (AUC = 0.687; bootstrap 95% CI: 0.618–0.755). At the 0.63 cut-off, sensitivity was 89.2%, specificity 47.3%, positive predictive value 54.6%, and negative predictive value 86.1%. In the primary multivariable model, Hb/RDW remained associated with later preeclampsia (adjusted OR per 0.1-unit increase = 1.45; 95% CI: 1.22–1.72; *p* < 0.001). However, Hb/RDW was not independently associated with severe preeclampsia among affected patients, fetal growth restriction, or composite maternal morbidity. VIF values ranged from 1.00 to 1.07. Secondary models for fetal growth restriction and composite maternal morbidity had borderline events-per-variable values and were interpreted as exploratory. **Conclusions:** First-trimester Hb/RDW was associated with later documented preeclampsia in this retrospective tertiary-care cohort, but its single-marker performance was modest and specificity was limited. The ratio should be interpreted as an inexpensive research marker of hematologic phenotype rather than a stand-alone screening, diagnostic, or management tool. Prospective multicenter validation with standardized first-trimester sampling and adjustment for hematinic and nutritional variables is required before clinical implementation.

## 1. Introduction

Preeclampsia is a multisystem hypertensive disorder of pregnancy characterized by abnormal placentation, endothelial dysfunction, angiogenic imbalance, inflammatory activation, and maternal organ involvement. It remains a major contributor to maternal and perinatal morbidity, particularly when disease occurs early or progresses to severe features [1,2,3].

The pathophysiology is not explained by a single pathway. Impaired trophoblastic invasion, placental hypoperfusion, oxidative stress, soluble antiangiogenic factor excess, and systemic endothelial injury interact with maternal constitutional and comorbid factors [4,5,6,7]. This biological heterogeneity explains why many candidate biomarkers show statistical association but fail to reach sufficient clinical performance for individual-level decision-making.

Complete blood count-derived indices are attractive in obstetric research because they are inexpensive, reproducible, and available from routine antenatal care. Red cell distribution width (RDW) has been examined as a marker related to erythrocyte heterogeneity, inflammation, oxidative stress, nutritional status, and endothelial dysfunction in pregnancy [8,9,10,11,12,13]. However, the literature on RDW and preeclampsia remains inconsistent, and RDW alone has not been established as a clinically actionable test.

Other blood count-derived and serum-based ratios, including the neutrophil-to-lymphocyte ratio, platelet-to-lymphocyte ratio, systemic immune–inflammatory indices, and fibrinogen-to-albumin ratio, have also been evaluated in preeclampsia with variable results [14,15,16]. These markers should be interpreted against the background of established multimodal first-trimester approaches that combine maternal factors, biophysical measures, and angiogenic biomarkers rather than rely on a single hematologic variable [17,18].

The hemoglobin-to-RDW (Hb/RDW) ratio combines oxygen-carrying capacity and erythrocyte size variability into one erythrocyte-derived index. It has been investigated mainly in non-obstetric inflammatory, cardiovascular, and oncologic settings [19,20,21]. Evidence regarding its role in preeclampsia is limited, and the clinical meaning of a higher or lower ratio may differ across populations depending on hemoconcentration, nutritional background, iron status, gestational timing, and case-mix.

Therefore, the present study evaluated the association between the first-trimester Hb/RDW ratio and later documented preeclampsia in a retrospective tertiary-care cohort. We also assessed discrimination, cut-off performance, relation to disease severity, association with maternal–neonatal outcomes, and the stability and interpretability of multivariable models. The study was deliberately framed as an association and discrimination analysis, not as validation of a prospective screening test.

## 2. Materials and Methods

### 2.1. Study Design, Setting, and Ethics

This retrospective cohort study was conducted at Şanlıurfa Training and Research Hospital, a tertiary referral center for high-risk obstetric care. Singleton pregnancies managed between January 2024 and December 2025 were screened through institutional electronic medical records.

Ethical approval was obtained from the Diyarbakır Gazi Yaşargil Training and Research Hospital Clinical Research Ethics Committee (Approval No. 72; Date: 2 March 2026). The approval covered retrospective evaluation of first-trimester hemoglobin and RDW parameters in relation to subsequent preeclampsia status and disease severity. The study was conducted in accordance with the Declaration of Helsinki and institutional ethical standards. Because anonymized retrospective clinical data were used, the requirement for written informed consent was waived by the ethics committee.

The clinical care period preceded ethics approval because the study was retrospective. No analysis was performed before approval. Data extraction, anonymization, statistical analysis, and manuscript preparation were initiated only after the ethics approval date.

### 2.2. Study Population

All consecutive singleton pregnancies evaluated during the study period were screened for eligibility. Exclusion criteria were multiple gestation, known hematologic disorder, chronic inflammatory disease, autoimmune disease, active infection at the index evaluation, malignancy, and incomplete clinical or laboratory records required for the study variables.

**Figure 1:**
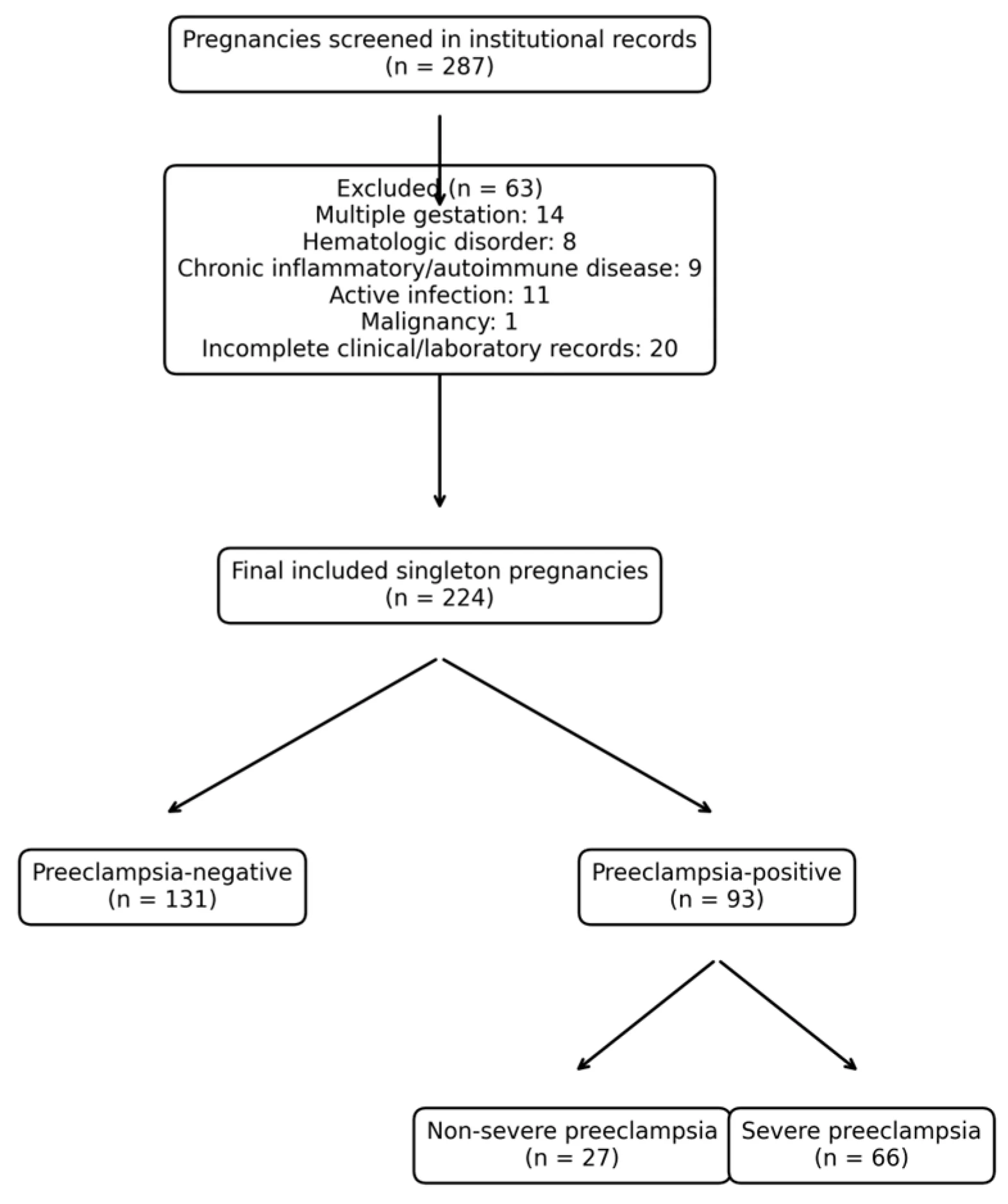
Patient selection flow diagram.

A total of 287 pregnancies were screened. After exclusion of 14 multiple gestations, 8 cases with hematologic disorders, 9 cases with chronic inflammatory or autoimmune disease, 11 cases with active infection, 1 case with malignancy, and 20 cases with incomplete clinical or laboratory records, 224 singleton pregnancies were included in the final analysis. The patient selection process is shown in Figure 1.

Participants were categorized as preeclampsia-negative (PE−) or preeclampsia-positive (PE+). Among the 93 PE+ patients, disease was further categorized as non-severe or severe preeclampsia.

### 2.3. Clinical Definitions and Outcomes

Preeclampsia was defined according to American College of Obstetricians and Gynecologists Practice Bulletin No. 222 as new-onset hypertension after 20 weeks of gestation accompanied by proteinuria and/or maternal end-organ dysfunction [7].

Severe preeclampsia was defined according to ACOG severe-feature criteria, including severe-range blood pressure, thrombocytopenia, impaired liver function with persistent symptoms or transaminase elevation, progressive renal insufficiency, pulmonary edema, new-onset cerebral or visual symptoms, HELLP syndrome, or eclampsia. When diagnostic overlap occurred, patients were classified according to the most severe clinical category documented during the index admission.

Fetal growth restriction (FGR) was defined as estimated fetal weight or birth weight below the 10th percentile for gestational age, or as a clinical diagnosis documented by the attending maternal–fetal medicine team in accordance with institutional practice. The primary outcome was later documented as preeclampsia. Secondary outcomes were severe preeclampsia among PE+ patients, FGR, neonatal death, and composite maternal adverse outcome.

Composite maternal adverse outcome included intensive care unit admission, maternal transfusion requirement, prolonged hospitalization, obstetric hemorrhage, rehospitalization, or maternal mortality. Because these components represent clinically heterogeneous events, component-level frequencies were reported in addition to the composite endpoint.

### 2.4. Data Collection, Anonymization, and Laboratory Assessment

Demographic characteristics, obstetric variables, laboratory values, and neonatal outcomes were obtained from institutional electronic medical records. Recorded variables included maternal age, gravida, gestational age at delivery, neonatal birth weight, Apgar scores, FGR, neonatal mortality, and maternal adverse outcomes.

Before analysis, each eligible record was assigned a study code and direct identifiers, including patient names, national identification numbers, hospital protocol numbers, addresses, and telephone numbers, were removed from the analysis file. The linkage key was not included in the statistical dataset. The anonymized dataset was stored on password-protected institutional computers accessible only to the study investigators.

Laboratory parameters were obtained from routine venous blood samples collected during first-trimester antenatal evaluation. For all included participants, the Hb/RDW ratio used in the analysis was calculated from a complete blood count obtained before clinical diagnosis of preeclampsia or before final classification as PE-negative. This timing was specified to distinguish temporal measurement from retrospective outcome ascertainment.

Samples were collected using standard hospital phlebotomy procedures and analyzed in the central laboratory with automated hematology and biochemistry analyzers. Complete blood count samples were processed immediately after collection and were not frozen. Because this was a retrospective clinical study, fasting status was not uniformly documented and could not be standardized.

Measured hematologic parameters included hemoglobin concentration, RDW, platelet count, neutrophil count, lymphocyte count, and monocyte count. Biochemical parameters included creatinine, albumin, aspartate aminotransferase (AST), and alanine aminotransferase (ALT). The Hb/RDW ratio was calculated as hemoglobin level (g/dL) divided by RDW percentage (%).

### 2.5. Statistical Analysis

All statistical analyses were performed using IBM SPSS Statistics for Windows, version 26.0 (IBM Corp., Armonk, NY, USA). Distribution normality was evaluated using histogram inspection and the Shapiro–Wilk test. Normally distributed variables were presented as mean ± standard deviation, whereas non-normally distributed variables were expressed as median (interquartile range). Categorical variables were presented as number and percentage.

Between-group comparisons were performed using the independent-samples t test or Mann–Whitney U test, as appropriate. Categorical variables were compared using Pearson’s chi-square test or Fisher’s exact test. ROC curve analysis was used to evaluate discrimination of first-trimester Hb/RDW for later documented preeclampsia. AUC and bootstrap 95% confidence intervals were calculated. The 0.63 cut-off was evaluated for sensitivity, specificity, positive predictive value (PPV), negative predictive value (NPV), accuracy, and likelihood ratios.

Multivariable logistic regression was used to assess the association between first-trimester Hb/RDW and later preeclampsia after adjustment for routinely available laboratory variables. Candidate covariates were selected a priori based on clinical relevance and routine availability. Creatinine, albumin, platelet count, and AST were included as contextual laboratory covariates to examine whether Hb/RDW retained association in the presence of other clinically relevant abnormalities; these variables were not interpreted as baseline causal confounders because they may partly reflect preeclampsia-related organ involvement. Hb/RDW was entered per 0.1-unit increase to improve clinical interpretability.

Multicollinearity was assessed using variance inflation factors (VIFs). Events per variable (EPV) were calculated for each logistic model to evaluate potential sparse-event bias and overfitting risk. Model calibration was explored using the Hosmer–Lemeshow goodness-of-fit test, and discrimination was summarized using model AUC and Brier score. Exploratory incremental discrimination models compared basic obstetric variables, organ-involvement laboratory covariates, and the addition of Hb/RDW.

No formal a priori sample-size calculation was conducted because all eligible cases within the approved retrospective period were analyzed. To address statistical adequacy, the precision of the primary ROC estimate was represented by the bootstrap 95% confidence interval, and model stability was evaluated using EPV. This approach was considered adequate for the primary association/discrimination analysis but not for definitive inference from secondary sparse-event models.

Missing clinical or laboratory data required for study variables were handled by complete-case analysis. No imputation was performed. Two-sided *p* values <0.05 were considered statistically significant.

## 3. Results

### 3.1. Study Population and Baseline Characteristics

A total of 224 singleton pregnancies were included in the final analysis (Figure 1). Of these, 131 women (58.5%) were classified as PE− and 93 women (41.5%) as PE+. Among PE+ patients, 27 (29.0%) had non-severe preeclampsia and 66 (71.0%) had severe preeclampsia.

Baseline maternal and obstetric characteristics are summarized in Table 1. Maternal age and gravida were comparable between groups. Pregnancies complicated by preeclampsia had significantly earlier gestational age at delivery and lower neonatal birth weight. FGR, lower Apgar scores, neonatal death, and composite maternal adverse outcome were more frequent in the PE+ group.

### 3.2. Laboratory Findings

**Figure 2:**
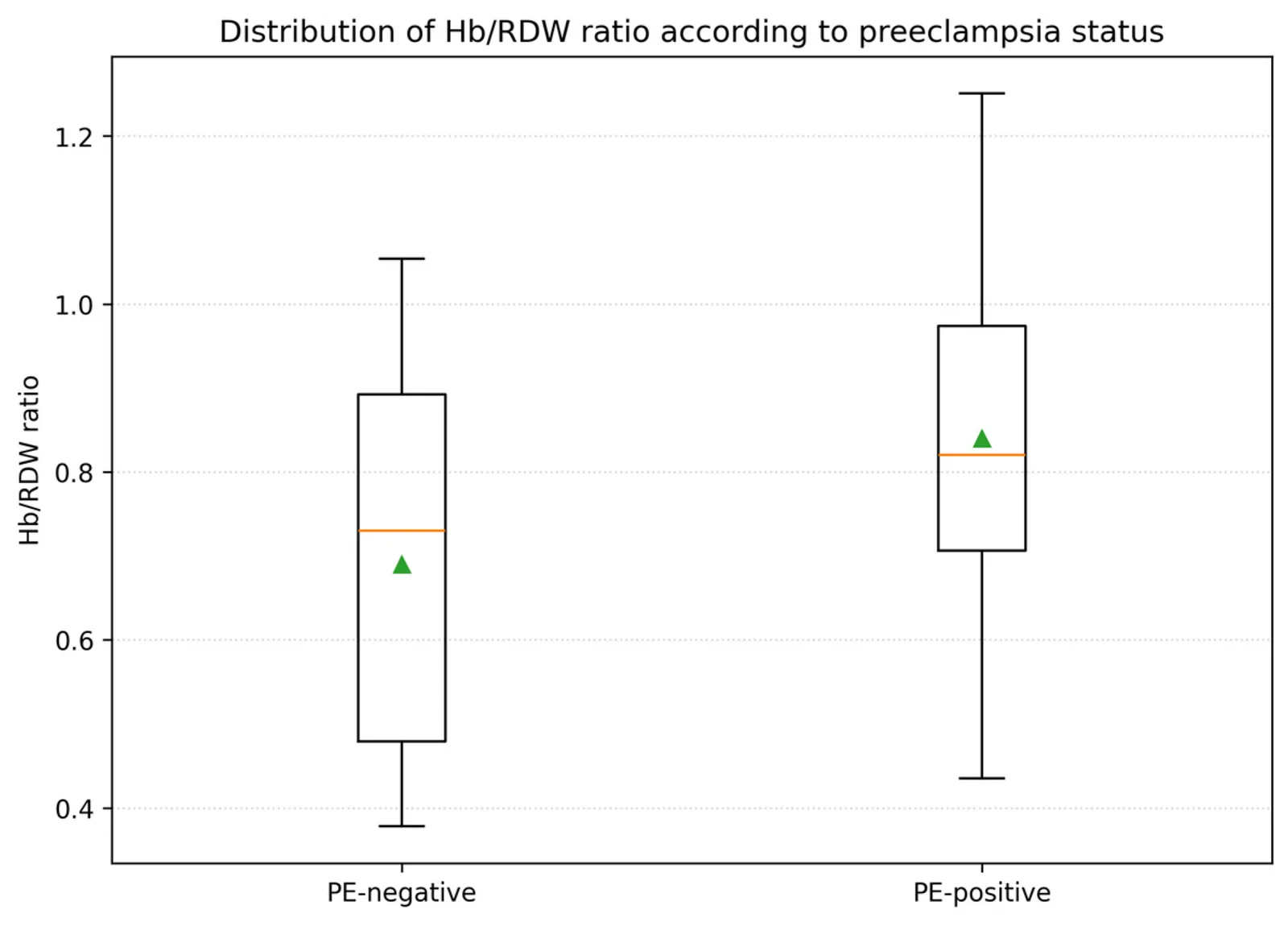
Distribution of Hb/RDW ratio according to preeclampsia status. The orange line represents the median, and the green triangle represents the mean.

Laboratory findings are summarized in Table 2. The Hb/RDW ratio was higher in PE+ pregnancies than in PE− pregnancies (0.84 ± 0.17 vs. 0.69 ± 0.21; *p* < 0.001). Hemoglobin was higher and RDW was lower in the PE+ group, resulting in an elevated Hb/RDW ratio. The distribution of Hb/RDW ratio values according to preeclampsia status is shown in Figure 2.

Creatinine and liver enzyme levels were higher in PE+ pregnancies, whereas albumin and platelet counts were lower. AST and ALT showed distributional skewness; therefore, these variables and related regression estimates were interpreted cautiously.

### 3.3. Disease Severity and Composite Outcome Components

Severity distribution among PE+ patients is presented in Table 3. Component-level analysis of the composite maternal adverse outcome is shown in Table 4. The most frequent component was prolonged hospitalization, followed by maternal transfusion requirement and intensive care unit admission. No maternal death occurred in either group.

### 3.4. ROC Analysis and Cut-Off Performance

**Figure 3:**
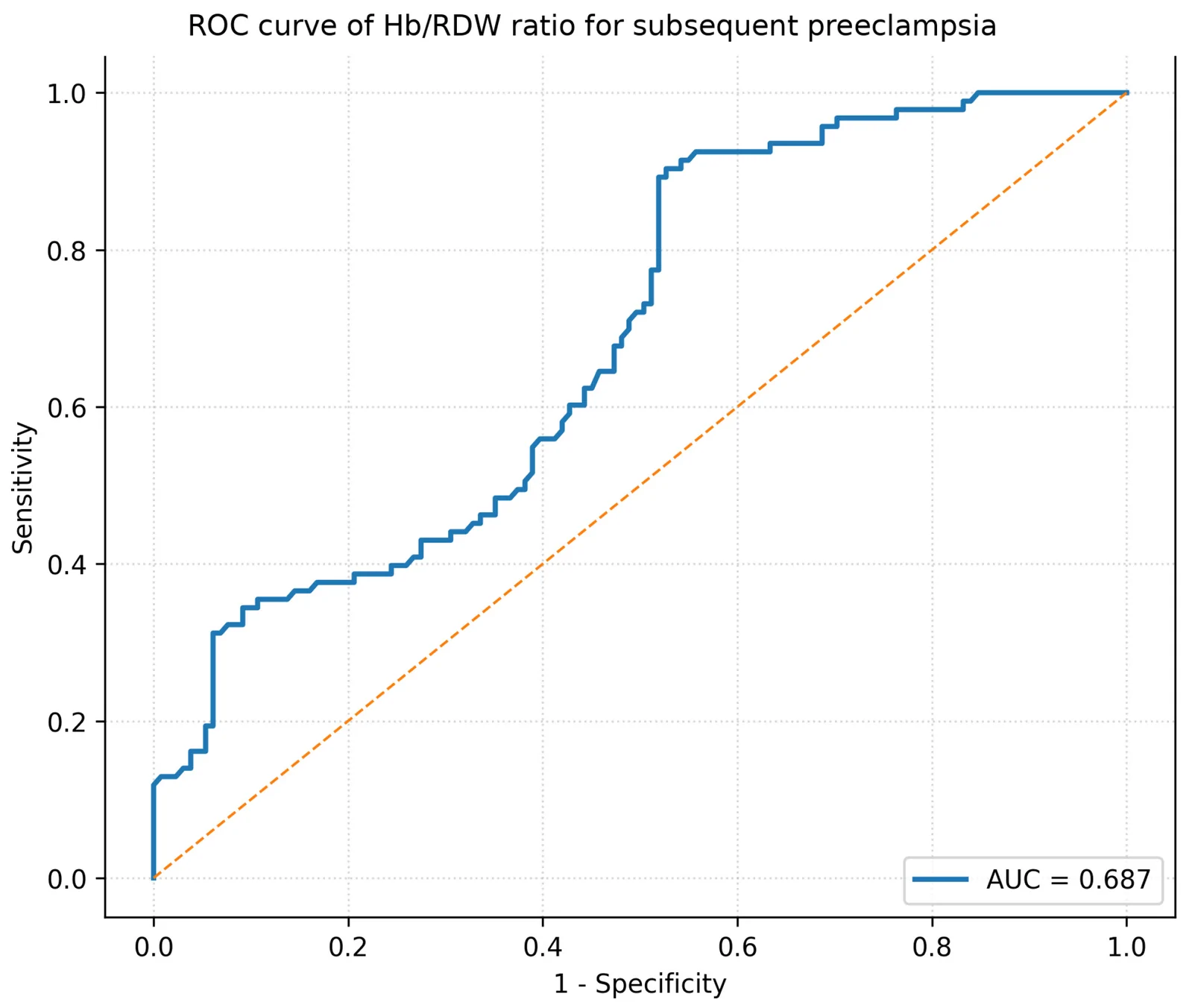
Receiver operating characteristic curve of first-trimester Hb/RDW ratio for subsequent preeclampsia.

ROC analysis demonstrated that the first-trimester Hb/RDW ratio had moderate discrimination for later documented preeclampsia (AUC = 0.687; bootstrap 95% CI: 0.618–0.755) (Table 5, Figure 3). At the 0.63 cut-off, sensitivity was 89.2%, specificity 47.3%, PPV 54.6%, NPV 86.1%, accuracy 64.7%, LR+ 1.69, and LR− 0.23. The high sensitivity was accompanied by poor specificity and a low positive likelihood ratio; therefore, the marker does not have sufficient stand-alone clinical performance.

### 3.5. Multivariable Regression and Model Diagnostics

In the primary multivariable model, first-trimester Hb/RDW remained associated with later preeclampsia after adjustment for creatinine, albumin, platelet count, and AST (adjusted OR per 0.1-unit increase = 1.45; 95% CI: 1.22–1.72; *p* < 0.001) (Table 6). Creatinine, albumin, and AST were also associated with preeclampsia, whereas platelet count was not.

Secondary multivariable models are summarized in Table 7. Among PE+ patients, Hb/RDW was not independently associated with severe preeclampsia. Hb/RDW was also not independently associated with FGR or composite maternal adverse outcome after adjustment. The large creatinine estimate in the composite maternal morbidity model indicated sparse-data instability and was not interpreted as a precise effect estimate.

Model diagnostics are presented in Table 8. VIF values ranged from 1.00 to 1.07, indicating no relevant multicollinearity among candidate predictors. EPV was acceptable for the primary preeclampsia model (18.6) but borderline for FGR (8.4) and composite maternal adverse outcome (5.8), supporting cautious interpretation of secondary models. The primary model showed no evidence of poor calibration by Hosmer–Lemeshow testing, but this does not substitute for external validation.

### 3.6. Correlation Analysis

Spearman correlation analysis is summarized in Table 9. Hb/RDW showed modest negative correlations with gestational age at delivery and neonatal birth weight, and weak positive correlations with creatinine and ALT. These correlations were small in magnitude and should be interpreted as descriptive rather than mechanistic.

## 4. Discussion

This study has three principal findings. First, a higher first-trimester Hb/RDW ratio was associated with later documented preeclampsia in a tertiary-care retrospective cohort. Second, the ratio showed only moderate single-marker discrimination and limited specificity. Third, Hb/RDW was not independently associated with severe preeclampsia among affected patients, FGR, or composite maternal morbidity after adjustment. These findings support a restrained interpretation: Hb/RDW may describe a hematologic phenotype observed before clinical diagnosis, but it does not provide sufficient evidence for stand-alone screening or management use.

The chronology of biomarker measurement is important. Hb/RDW was calculated from first-trimester complete blood count data obtained before clinical diagnosis of preeclampsia. Nevertheless, outcome status was ascertained retrospectively after the pregnancy course was known. For this reason, the present analysis should be understood as a retrospective association and discrimination study. It does not prove prospective predictive validity, clinical utility, or benefit from changing surveillance or preventive treatment on the basis of Hb/RDW.

The clinical performance metrics are central to interpretation. The AUC of 0.687 indicates moderate discrimination, and specificity of 47.3% at the evaluated cut-off would generate many false-positive classifications in clinical practice. The positive likelihood ratio of 1.69 is too small to meaningfully rule in future disease, whereas the negative likelihood ratio of 0.23 suggests only partial exclusionary value. These results are inferior to validated first-trimester multimodal approaches that incorporate maternal factors, biophysical parameters, and angiogenic biomarkers, and they should not be positioned as an alternative to established screening or prevention frameworks [17,18].

The biological interpretation of a higher Hb/RDW ratio requires caution. Preeclampsia is associated with plasma volume contraction, endothelial dysfunction, renal involvement, and systemic inflammation [1,2,3,4,5,6,7]. A higher hemoglobin concentration may reflect hemoconcentration in some patients, whereas RDW is influenced by anisocytosis, oxidative stress, inflammation, iron status, folate and vitamin B12 status, and nutritional background [8,9,10,11,12,13]. Therefore, the ratio may change because of either numerator or denominator effects and should not be interpreted as a direct equivalent of RDW.

The finding of lower RDW values in the PE+ group differs from several reports and meta-analyses describing higher RDW in preeclampsia [9,11,12,13]. This discordance is scientifically relevant rather than merely inconvenient. Potential explanations include first-trimester sampling, referral-center case-mix, a high proportion of severe disease, local nutritional and iron-status patterns, exclusion of active inflammatory conditions, and laboratory-method differences. Because ferritin, vitamin B12, folate, smoking status, and detailed nutritional variables were not systematically available, the present dataset cannot determine whether the RDW direction reflects disease biology, population characteristics, or residual confounding.

The multivariable findings should also be interpreted within the design of the model. Creatinine, albumin, platelet count, and AST are clinically relevant in preeclampsia evaluation, but they may be manifestations or consequences of disease-related organ involvement rather than true baseline confounders. Their inclusion was intended to test whether Hb/RDW retained association in the presence of routine laboratory abnormalities, not to establish causality. Accordingly, the phrase independent association should be read statistically and contextually, not mechanistically.

The secondary models were less stable than the primary model. Although multicollinearity was not evident, the composite maternal morbidity model had a low EPV and produced a very large creatinine estimate with a wide confidence interval. Such estimates commonly occur in sparse-event settings and should not be interpreted as precise effect sizes. The absence of an independent association between Hb/RDW and severe disease or maternal morbidity is clinically informative because it suggests that the ratio may not capture the organ-injury phenotype that defines clinically severe preeclampsia.

External validity is limited by the selected tertiary-care population. More than 70% of PE+ patients had severe preeclampsia, and neonatal death and FGR were substantially more frequent in the PE+ group. This enrichment increases the likelihood of detecting laboratory differences but reduces generalizability to unselected low-risk antenatal populations. The present results therefore should not be extrapolated to primary-care screening without prospective validation in broader cohorts.

This study also has strengths. The biomarker was obtained from routine first-trimester complete blood count data, the temporal relationship between sampling and later disease classification was explicitly clarified, standardized diagnostic criteria were used, component-level maternal morbidity was reported, ROC confidence intervals and cut-off metrics were provided, and model diagnostics including VIF, EPV, calibration, and exploratory incremental discrimination were added. These elements improve transparency and help separate statistical association from clinical applicability.

Several limitations remain. The retrospective single-center design limits causal inference and may introduce selection bias. Laboratory parameters were measured at a single first-trimester time point, preventing assessment of longitudinal hematologic trajectories. Fasting status, iron indices, ferritin, vitamin B12, folate, smoking status, socioeconomic variables, medication exposure, and nutritional background were not systematically available. The tertiary referral setting and high proportion of severe disease limit generalizability. Finally, the modest AUC, low specificity, and borderline EPV of secondary models mean that Hb/RDW should be considered hypothesis-generating rather than clinically actionable.

Future research should evaluate Hb/RDW prospectively using standardized sampling windows, gestational age-specific reference distributions, and adjustment for hematinic and nutritional variables. The key question is not whether Hb/RDW differs between groups, but whether it adds reproducible incremental value beyond established clinical, Doppler, and angiogenic parameters in externally validated models. Until such evidence is available, Hb/RDW should remain a low-cost exploratory marker rather than a clinical decision tool.

## 5. Conclusions

The first-trimester Hb/RDW ratio was associated with later documented preeclampsia in this retrospective tertiary-care cohort. However, discrimination was moderate, specificity was limited, and Hb/RDW was not independently associated with severe preeclampsia, FGR, or composite maternal morbidity. The results should be interpreted as hypothesis-generating evidence of hematologic association, not as validation of a stand-alone screening, diagnostic, or management tool. Prospective multicenter studies with standardized first-trimester sampling and adjustment for hematinic and nutritional variables are required before clinical implementation.

## Figures and Tables

**Table 1:** Baseline maternal and obstetric characteristics according to preeclampsia status.

Variable	PE− (*n* = 131)	PE+ (*n* = 93)	*p* Value
Maternal age (years)	27.21 ± 5.47	29.29 ± 6.72	0.061
Gravida	3.00 (2.00–4.00)	3.00 (1.00–5.00)	0.447
Gestational week at delivery	36.95 ± 1.70	32.16 ± 3.23	<0.001
Birth weight (g)	3010.53 ± 495.13	1835.16 ± 553.84	<0.001
FGR, *n* (%)	3 (2.3%)	39 (41.9%)	<0.001
Apgar score at 1 min	8.00 (8.00–8.00)	6.00 (4.00–6.00)	<0.001
Apgar score at 5 min	9.00 (9.00–10.00)	8.00 (6.00–8.00)	<0.001
Neonatal death, *n* (%)	1 (0.8%)	30 (32.3%)	<0.001
Composite maternal adverse outcome, *n* (%)	2 (1.5%)	27 (29.0%)	<0.001

Note. Values are presented as mean ± standard deviation, median (interquartile range), or *n* (%), as appropriate. FGR: fetal growth restriction; PE: preeclampsia.

**Table 2:** Laboratory parameters according to preeclampsia status.

Variable	Reference Interval *	PE− (*n* = 131)	PE+ (*n* = 93)	*p* Value
Hemoglobin (g/dL)	11.0–16.0	10.95 ± 1.69	11.96 ± 1.50	<0.001
RDW (%)	11.5–14.5	16.67 ± 3.06	14.69 ± 2.29	<0.001
Hb/RDW ratio	Not established	0.69 ± 0.21	0.84 ± 0.17	<0.001
Platelet (×10^9^/L)	150–400	245.55 ± 55.83	221.51 ± 80.28	0.014
Creatinine (mg/dL)	0.50–0.90	0.54 ± 0.17	0.68 ± 0.19	<0.001
Albumin (g/L)	35–50	35.66 ± 4.35	32.37 ± 4.13	<0.001
AST (U/L)	0–35	21.86 ± 7.39	37.57 ± 59.00	0.003
ALT (U/L)	0–35	11.20 ± 4.14	21.93 ± 36.40	0.009

Note. * Reference intervals are institutional routine laboratory reference intervals and may vary by assay, gestational age, and local laboratory calibration. Hb/RDW: hemoglobin-to-red cell distribution width ratio; RDW: red cell distribution width; AST: aspartate aminotransferase; ALT: alanine aminotransferase.

**Table 3:** Severity distribution among patients with preeclampsia.

Severity Category	*n*	% Among PE+ Patients
Non-severe preeclampsia	27	29.0
Severe preeclampsia	66	71.0
Total	93	100.0

Note. Severe preeclampsia was defined according to ACOG severe-feature criteria.

**Table 4:** Individual components of the composite maternal adverse outcome.

Component	PE− (*n* = 131)	PE+ (*n* = 93)	Total (*N* = 224)
Any composite maternal adverse outcome	2 (1.5%)	27 (29.0%)	29 (12.9%)
Intensive care unit admission	0 (0.0%)	10 (10.8%)	10 (4.5%)
Maternal transfusion requirement	1 (0.8%)	12 (12.9%)	13 (5.8%)
Prolonged hospitalization	2 (1.5%)	23 (24.7%)	25 (11.2%)
Obstetric hemorrhage	1 (0.8%)	7 (7.5%)	8 (3.6%)
Rehospitalization	0 (0.0%)	5 (5.4%)	5 (2.2%)
Maternal mortality	0 (0.0%)	0 (0.0%)	0 (0.0%)

Note. Components are not mutually exclusive; therefore, component counts may exceed the number of patients with any composite event.

**Table 5:** ROC and cut-off performance of first-trimester Hb/RDW ratio for subsequent preeclampsia.

Metric	Value
AUC	0.687
Bootstrap 95% CI	0.618–0.755
Used cut-off	0.63
Sensitivity	89.2%
Specificity	47.3%
PPV	54.6%
NPV	86.1%
Accuracy	64.7%
LR+	1.69
LR−	0.23
True positives/false negatives	83/10
True negatives/false positives	62/69

Note. The analysis evaluates discrimination for later documented preeclampsia in a retrospective cohort based on first-trimester complete blood count data. AUC: area under the curve; PPV: positive predictive value; NPV: negative predictive value; LR: likelihood ratio.

**Table 6:** Multivariable logistic regression analysis for subsequent preeclampsia.

Predictor	Adjusted OR	95% CI	*p* Value
Hb/RDW ratio, per 0.1-unit increase	1.45	1.22–1.72	<0.001
Creatinine, per 1 mg/dL increase	36.82	5.55–244.24	<0.001
Albumin, per 1 g/L increase	0.85	0.78–0.92	<0.001
Platelet, per 1 × 10^9^/L increase	1.00	0.99–1.00	0.145
AST, per 1 U/L increase	1.03	1.01–1.04	0.007

Note. Adjusted odds ratios were obtained from a multivariable logistic regression model including Hb/RDW ratio, creatinine, albumin, platelet count, and AST. Hb/RDW was scaled per 0.1-unit increase. Laboratory covariates were used for contextual adjustment and were not interpreted as baseline causal confounders.

**Table 7:** Secondary multivariable logistic regression analyses.

Outcome	Predictor	Adjusted OR	95% CI	*p* Value
Severe PE among PE+ (*N* = 93; events = 66)	Hb/RDW per 0.1	0.89	0.67–1.17	0.407
	Creatinine per 1 mg/dL	2.10	0.17–25.49	0.561
	Albumin per 1 g/L	0.96	0.85–1.08	0.457
	Platelet per 1 × 10^9^/L	1.01	1.00–1.01	0.077
	AST per 1 U/L	1.01	0.99–1.02	0.411
FGR (*N* = 224; events = 42)	Hb/RDW per 0.1	1.00	0.84–1.19	0.990
	Creatinine per 1 mg/dL	5.93	0.85–41.16	0.072
	Albumin per 1 g/L	0.88	0.80–0.96	0.003
	Platelet per 1 × 10^9^/L	1.00	0.99–1.00	0.400
	AST per 1 U/L	1.01	1.00–1.02	0.020
Composite maternal adverse outcome (*N* = 224; events = 29)	Hb/RDW per 0.1	1.17	0.87–1.58	0.310
	Creatinine per 1 mg/dL	1995.47	61.98–64,241.27	<0.001
	Albumin per 1 g/L	0.78	0.66–0.91	0.001
	Platelet per 1 × 10^9^/L	1.00	1.00–1.01	0.366
	AST per 1 U/L	1.06	1.03–1.08	<0.001

Note. All models included Hb/RDW ratio, creatinine, albumin, platelet count, and AST. The severe preeclampsia model was restricted to patients with preeclampsia. Composite-outcome estimates, especially creatinine, should be interpreted cautiously because of sparse events and model instability.

**Table 8:** Model diagnostics and exploratory incremental discrimination analyses.

Domain	Metric/Model	Value	Interpretation
Multicollinearity	VIF range	1.00–1.07	No relevant multicollinearity
EPV	Preeclampsia model	18.6	Generally acceptable
EPV	Severe PE among PE+	13.2	Generally acceptable
EPV	FGR model	8.4	Borderline; sparse-event caution
EPV	Composite morbidity model	5.8	Borderline; sparse-event caution
Calibration	Primary model Hosmer-Lemeshow p	0.919	No evidence of poor calibration
Model performance	Primary model AUC/Brier score	0.845/0.159	Exploratory retrospective model
Incremental AUC	Age + gravida	0.591 (95% CI 0.510–0.669)	Basic comparator
Incremental AUC	Age + gravida + Hb/RDW	0.711 (95% CI 0.645–0.778)	Hb/RDW increased discrimination vs. basic model
Incremental AUC	Organ-lab covariates without Hb/RDW	0.803 (95% CI 0.743–0.858)	Laboratory comparator
Incremental AUC	Organ-lab covariates + Hb/RDW	0.845 (95% CI 0.792–0.891)	Exploratory incremental discrimination

Note. Incremental AUC models evaluate discrimination for later documented preeclampsia using retrospective first-trimester data and should be interpreted as exploratory. EPV: events per variable; VIF: variance inflation factor; AUC: area under the curve.

**Table 9:** Spearman correlation analysis between Hb/RDW ratio and clinical-laboratory variables.

Variable	Spearman *ρ* with Hb/RDW
Gestational week at delivery	−0.18
Birth weight	−0.23
Creatinine	0.18
Platelet count	−0.10
Albumin	0.14
AST	0.01
ALT	0.17

Note. Spearman rank correlation coefficients were calculated to assess relationships between Hb/RDW ratio and clinical-laboratory variables.

## Data Availability

The datasets generated and analyzed during the current study are not publicly available because they contain de-identified clinical data subject to institutional and national privacy regulations, but are available from the corresponding author upon reasonable request and with appropriate institutional permissions.
